# Extracellular Water to Total Body Water Ratio in Viral Liver Diseases: A Study Using Bioimpedance Analysis

**DOI:** 10.3390/nu10081072

**Published:** 2018-08-12

**Authors:** Hiroki Nishikawa, Kazunori Yoh, Hirayuki Enomoto, Noriko Ishii, Yoshinori Iwata, Chikage Nakano, Ryo Takata, Takashi Nishimura, Nobuhiro Aizawa, Yoshiyuki Sakai, Naoto Ikeda, Kunihiro Hasegawa, Tomoyuki Takashima, Hiroko Iijima, Shuhei Nishiguchi

**Affiliations:** Division of Hepatobiliary and Pancreatic disease, Department of Internal Medicine, Hyogo College of Medicine, Nishinomiya, Hyogo 663-8501, Japan; mm2wintwin@ybb.ne.jp (K.Y.); enomoto@hyo-med.ac.jp (H.E.); ishinori1985@yahoo.co.jp (N.I.); yo-iwata@hyo-med.ac.jp (Y.I.); chikage@hyo-med.ac.jp (C.N.); chano_chano_rt@yahoo.co.jp (R.T.); tk-nishimura@hyo-med.ac.jp (T.N.); nobu23hiro@yahoo.co.jp (N.A.); sakai429@hyo-med.ac.jp (Y.S.); nikeneko@hyo-med.ac.jp (N.I.); hiro.red1230@gmail.com (K.H.); tomo0204@yahoo.co.jp (T.T.); hiroko-i@hyo-med.ac.jp (H.I.); nishiguc@hyo-med.ac.jp (S.N.)

**Keywords:** extracellular water, total body water, hepatitis B virus, hepatitis C virus, bioimpedance analysis

## Abstract

Aims: To investigate the relationship between extracellular water to total body water ratio (ECW/TBW) in bioimpedance analysis (BIA) and clinical parameters in hepatitis viruses related to liver diseases. Methods: ECW/TBW was compared in patients with hepatitis B virus (HBV, *n* = 85) and hepatitis C virus (HCV, *n* = 440) related liver diseases. We also examined factors linked to mild to severe overhydrated state (ECW/TBW ≥0.4). Results: The median ECW/TBW in the HCV group was 0.388 (range, 0.365–0.433), while that in the HBV group was 0.381 (range, 0.363–0.425) (*p* < 0.0001). In all cases (*n* = 525), for predicting F3 or more, ECW/TBW yielded the area under the receiver operating characteristics (AUROC, 0.74912) and for predicting F4, ECW/TBW yielded the AUROC (0.75517). Multivariate analysis showed that age, prothrombin time, serum albumin, and alanine aminotransferase were significant factors linked to ECW/TBW ≥0.4. In patients with FIB-4 index <2, ECW/TBW in the HCV group was significantly higher than that in the HBV group (*p* = 0.0188), while in patients with 2 ≤ FIB-4 index <4 and FIB-4 index ≥4, the difference in the two groups did not reach significance. Conclusion: ECW/TBW can be different according to hepatitis viruses. Overhydrated status can easily occur in the HCV group even in the non-LC status compared with the HBV group.

## 1. Introduction

Approximately 50 to 70% of the body weight in a healthy person is water. It carries the ingested nutrients to the cells of the body and discharges the waste products to the outside, which suggests that it plays a role in transportation [1,2,3]. Body water is composed of intracellular water and extracellular water present in blood and interstitium, and when its equilibrium worsens, an edematous state tends to appear [1,2,3]. In cases of healthy persons, extracellular body water (EBW) to total body water (TBW) ratio (EBW/TBW) can be maintained at a constant value (EBW/TBW = 0.38) [1,2,3,4].

The significant relationship between fluid imbalance and clinical outcomes was found in studies investigating patients with conditions such as chronic obstructive pulmonary disease, acute heart failure, chronic liver diseases, HIV, renal disorders, and patients receiving peritoneal dialysis [5,6,7,8,9,10,11]. extracellular fluid (ECF) excess is a common condition in advanced liver cirrhosis (LC) patients with massive ascites [12,13]. Patients with LC and a first onset of ascites have a probability of overall survival of 85% during the first year and around 50% at 5 years [13,14,15,16]. Thus, identifying liver disease patients with overhydrated state in an earlier stage may be clinically of importance. In the current Japanese guidelines for LC, the administration of spironolactone has been recommended for LC patients with small ascites as a first line treatment [17].

Bioelectrical impedance analysis (BIA) has been introduced as a rapid, non-invasive, reproducible, easy to perform, and safe technique for the analysis of body composition including fat, muscle, and water [18]. In our previous studies using data for BIA, we have demonstrated clinical usefulness of BIA in liver disease patients [18,19,20].

However, currently, there is no reliable data regarding ECW/TBW in BIA in chronic hepatitis B virus (HBV) or hepatitis C virus (HCV) related liver diseases, especially those in the stage of non-LC. The practical fluid management in patients with chronic HBV or HCV related liver diseases involves the proper evaluation of fluid status in such patients.

The objective of this study was to investigate the relationship between ECW/TBW in BIA and other clinical parameters comparing HBV and HCV related chronic liver diseases.

## 2. Patients and Methods

### 2.1. Study Design

In this retrospective study, we analyzed a total of 525 patients with HBV related liver disease (the HBV group, *n* = 85) and HCV related liver disease (the HCV group, *n* = 440) who were admitted to the Division of Hepatobiliary and Pancreatic disease, Department of Internal Medicine, Hyogo College of Medicine, Hyogo, Japan between February 2006 and November 2015, and were assessed using BIA. Patients with massive ascites were excluded from this analysis because body composition analyses can be challenging in LC patients with severe fluid retention, that is, body weight, body mass index (BMI), and skeletal muscle mass index (SMI) in BIA may be overestimated in patients with massive ascites [20]. Hepatocellular carcinoma patients and HBV and HCV confection patients were also excluded from analysis. In the HBV group, all patients had detection of HB surface antigen for more than six months and there was no evidence of concurrent HCV infection, and no clear evidence of drug-induced or alcoholic liver disease, and 40 patients (47.1%) received previous antiviral therapies such as interferon therapies and nucleoside analogue therapies. In the HCV group, all patients had detection of HCV antibody and there was no evidence of concurrent HBV infection, and no clear evidence of drug-induced or alcoholic liver disease, and 226 patients (51.1%) received previous antiviral therapies such as interferon-based therapies and direct acting antiviral therapies. Skeletal muscle mass index in BIA was defined as “appendicular skeletal muscle mass/height (m)^2^” [21].

The diagnosis of LC was made on the basis of clinical data, including liver biopsy samples, laboratory tests, clinical features of portal hypertension, and/or medical imaging such as computed tomography. In non-LC patients, the degree of liver fibrosis (F0 to F3) was determined using liver biopsy samples.

We examined the relationship between ECW/TBW and other clinical parameters comparing the HBV group and the HCV group. According to the concept that excessive ECW results in edematous state, ECF status was defined as the ECW-to-TBW ratio (ECW/TBW), and ECF excess was classified as follows: mild overhydrated state (ECW/TBW 0.390–0.399) and moderate to severe overhydrated state (ECW/TBW ≥0.400) (Biospace Co. Ltd., Seoul, Korea) [4]. We also examined factors linked to mild to severe overhydrated state (ECW/TBW ≥0.4) using unilabiate and multivariate analyses [4].

The study protocol strictly complied with all provisions of the Declaration of Helsinki and was approved by the ethics committee of Hyogo College of Medicine, Nishinomiya, Hyogo, Japan (approval No. 2117). All patients gave written informed consent.

### 2.2. Statistical Analysis

For quantitative parameters, the statistical analysis between groups was performed using Student’s *t* test, Mann-Whitney *u* test, Kruskal-Wallis test, Fisher’s exact test or Spearman’s rank correlation coefficient *r_s_* as applicable. Parameters with *p* value < 0.05 in the unilabiate analysis were entered into the multivariate analysis utilizing the logistic regression analysis. In the multivariate analyses, significant variables in the unilabiate analyses were changed to dichotomous covariates using each median value. Receiver operating characteristics (ROC) curve analysis and area under the ROC curve (AUROC) results were presented along with the corresponding optimal cutoff point that maximized the sum of specificity and sensitivity, sensitivity and specificity. Data were expressed as median (range) unless otherwise stated. Statistically significance was defined as *p* < 0.05. Statistical analysis was performed with the JMP 13 (SAS Institute Inc., Cary, NC, USA).

## 3. Results

### 3.1. Patient Characteristics

**Figure 1:**
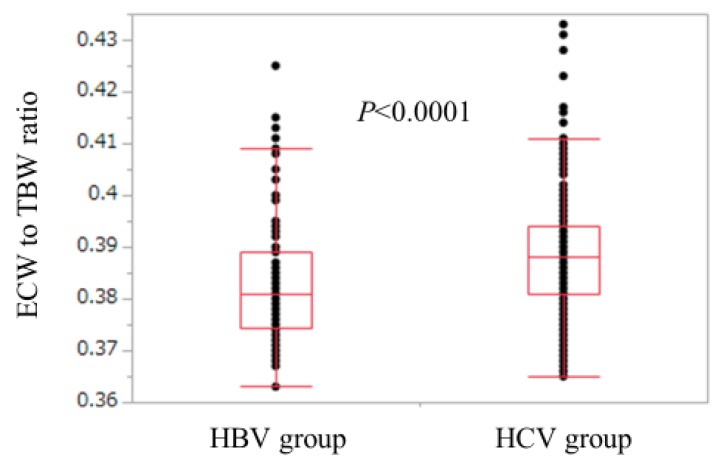
Comparison of ECW/TBW in the HBV group and the HCV group. ECW/TBW in the HCV group ranged from 0.365 to 0.433 (median, 0.388), while that in the HBV group ranged from 0.363 to 0.425 (median, 0.381). (*p* < 0.0001). HBV: hepatitis B virus; HCV: hepatitis C virus; ECW: extracellular water; TBW: total body water.

Baseline characteristics in all cases (*n* = 525), the HCV group (*n* = 440), and the HBV group (*n* = 85) are shown in Table 1. For the comparison of the HCV group and the HBV group, age (*p* < 0.0001), ECW/TBW (*p* < 0.0001), the proportion of LC (*p* = 0.0394), aspartate aminotransferase (AST) (*p* < 0.0001), alanine aminotransferase (ALT) (*p* = 0.0304), and FIB-4 index (*p* < 0.0001) in the HCV group were significantly higher than those in the HBV group, while SMI (*p* = 0.0099), serum albumin (*p* = 0.0017), platelet count (*p* = 0.0022), serum creatinine (*p* = 0.0021), total cholesterol (*p* = 0.010), and branched-chain amino acid to tyrosine ratio (BTR) (*p* = 0.0002) in the HCV group were significantly lower than those in the HBV group (Table 1). The ECW/TBW in the HCV group ranged from 0.365 to 0.433 (median, 0.388), while that in the HBV group ranged from 0.363 to 0.425 (median, 0.381) (Figure 1).

### 3.2. ECW/TBW According to Liver Fibrosis Stage and ROC Analyses for F3 or More and F4 in All Cases

**Figure 2:**
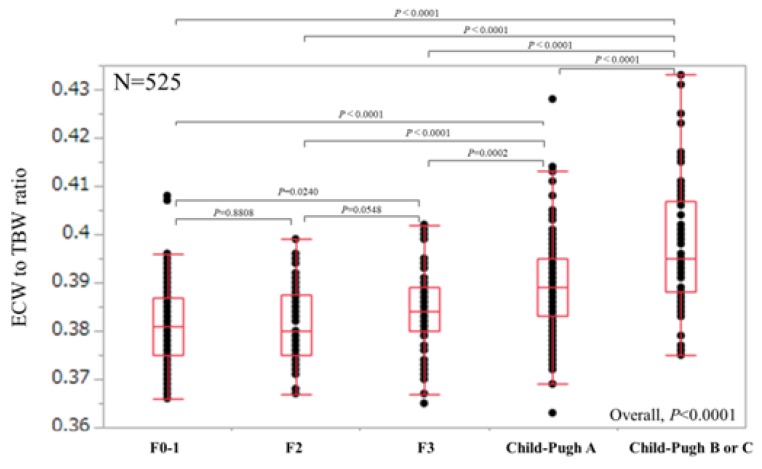
ECW/TBW according to liver fibrosis stage for all cases (*n* = 525). The median (range) ECW/TBW in each liver fibrosis stage were: 0.381 (0.366–0.408) in F0-1 (*n* = 106), 0.380 (0.367–0.399) in F2 (*n* = 53), 0.384 (0.365–0.402) in F3 (*n* = 64), 0.389 (0.363–0.428) in Child-Pugh A (*n* = 226) and 0.395 (0.375–0.433) in Child-Pugh B or C (*n* = 76) (*p* values; 0.8808 in F0-1 and F2, 0.0548 in F2 and F3, 0.0002 in F3 and Child-Pugh A, <0.0001 in Child-Pugh A and Child-Pugh B or C, 0.0240 in F0-1 and F3, <0.0001 in F2 and Child-Pugh A, <0.0001 in F3 and Child-Pugh B or C, <0.0001 in F0-1 and Child-Pugh A, <0.0001 in F2 and Child-Pugh B or C and <0.0001 in F0-1 and Child-Pugh B or C, overall significance *p* < 0.0001). ECW: extracellular water; TBW: total body water.

**Figure 3:**
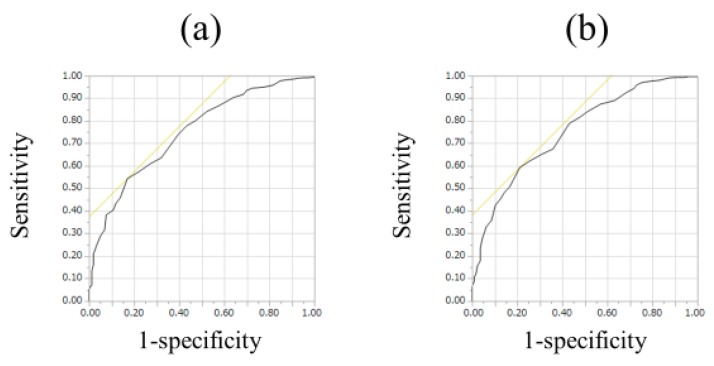
ROC analyses for F3 or more and F4 in all cases (*n* = 525). (**a**) For predicting F3 or more, ECW/TBW yielded the AUROC with a level of 0.74912 (optimal cutoff point 0.389, sensitivity 54.37%, and specificity 83.02%). (**b**) For predicting F4, ECW/TBW yielded the AUROC with a level of 0.75517 (optimal cutoff point 0.389, sensitivity 59.27%, and specificity 78.92%).

For all cases (*n* = 525), the median (range) ECW/TBW in each liver fibrosis stage were: 0.381 (0.366–0.408) in F0-1 (*n* = 106), 0.380 (0.367–0.399) in F2 (*n* = 53), 0.384 (0.365–0.402) in F3 (*n* = 64), 0.389 (0.363–0.428) in Child-Pugh A (*n* = 226), and 0.395 (0.375–0.433) in Child-Pugh B or C (*n* = 76) (*p* values; 0.8808 in F0-1 and F2, 0.0548 in F2 and F3, 0.0002 in F3 and Child-Pugh A, <0.0001 in Child-Pugh A and Child-Pugh B or C, 0.0240 in F0-1 and F3, <0.0001 in F2 and Child-Pugh A, <0.0001 in F3 and Child-Pugh B or C, <0.0001 in F0-1 and Child-Pugh A, <0.0001 in F2 and Child-Pugh B or C and <0.0001 in F0-1 and Child-Pugh B or C, overall significance *p* < 0.0001) (Figure 2). For predicting F3 or more, ECW/TBW yielded the AUROC with a level of 0.74912 (optimal cutoff point 0.389, sensitivity 54.37% and specificity 83.02%) and for predicting F4, ECW/TBW yielded the AUROC with a level of 0.75517 (optimal cutoff point 0.389, sensitivity 59.27%, and specificity 78.92%) (Figure 3a,b).

### 3.3. Relationship between ECW/TBW and Other Clinical Parameters

The relationships between ECW/TBW and other clinical parameters for all cases are demonstrated in Table 2. Significant variables with positive correlations with ECW/TBW were age and FIB-4 index. Significant variables with negative correlation with ECW/TBW were SMI, serum albumin, prothrombin time (PT), platelet count, serum creatinine, total cholesterol, triglyceride, ALT, and BTR. The *r_s_* values and *p* values of those factors are listed in Table 2.

### 3.4. Comparison of ECW/TBW in the HBV Group and the HCV Group According to Liver Fibrosis Stage (Non-LC, Child-Pugh A and Child-Pugh B or C)

We compared ECW/TBW in the HBV group and the HCV group in non-LC (F0 to F3), Child-Pugh A and Child-Pugh B or C patients.

**Figure 4:**
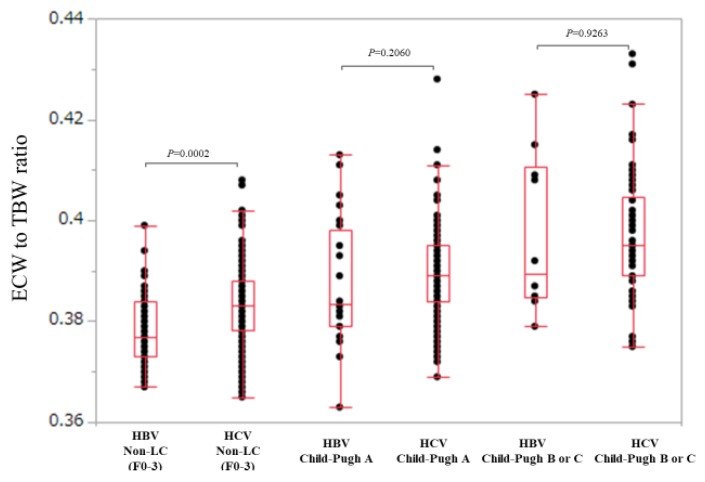
Comparison of ECW/TBW in the HBV group and the HCV group according to liver fibrosis stage (non-LC, Child-Pugh A and Child-Pugh B or C). In non-LC patients, ECW/TBW in the HCV group (*n* = 176) was significantly higher than that in the HBV group (*n* = 47) (median (range): 0.377 (0.367–0.399) in the HBV group and 0.383 (0.365–0.408) in the HCV group, *p* = 0.0002). In Child-Pugh A patients, ECW/TBW in the HCV group (*n* = 198) was not significantly higher than that in the HBV group (*n* = 28) (median (range): 0.380 (0.363–0.413) in the HBV group and 0.390 (0.369-0.428) in the HCV group, *p* = 0.2060). In Child-Pugh B or C patients, ECW/TBW in the HCV group (*n* = 66) was not significantly higher than that in the HBV group (*n* = 10) (median (range): 0.390 (0.379–0.425) in the HBV group and 0.395 (0.375–0.433) in the HCV group, *p* = 0.9263).

In non-LC patients (*n* = 47 and 176 in the HBV and HCV groups), ECW/TBW in the HCV group was significantly higher than that in the HBV group (median (range): 0.377 (0.367–0.399) in the HBV group and 0.383 (0.365–0.408) in the HCV group, *p* = 0.0002) (Figure 4).

In Child-Pugh A patients (*n* = 28 and 198 in the HBV and HCV groups), ECW/TBW in the HCV group was not significantly higher than that in the HBV group (median (range): 0.380 (0.363–0.413) in the HBV group and 0.390 (0.369–0.428) in the HCV group, *p* = 0.2060) (Figure 4).

Similarly, in Child-Pugh B or C patients (*n* = 10 and 66 in the HBV and HCV groups), ECW/TBW in the HCV group was not significantly higher than that in the HBV group (median (range): 0.390 (0.379–0.425) in the HBV group and 0.395 (0.375–0.433) in the HCV group, *p* = 0.9263) (Figure 4).

### 3.5. Unilabiate and Multivariate Analyses of Factors Associated with ECW/TBW ≥0.4

Unilabiate analysis identified eight factors to be significantly associated with ECW/TBW ≥0.4 (*p* < 0.05): age, serum albumin, PT, total cholesterol, triglyceride, ALT, BTR, and FIB-4 index (Table 3). Multivariate analysis for the seven factors (FIB-4 index was excluded because it includes age and ALT [22,23,24,25]) showed that age, PT, serum albumin, and ALT were significant factors linked to ECW/TBW ≥0.4 (Table 4). Hazard ratios and 95% confidence intervals of these factors are listed in Table 4.

### 3.6. Comparison of ECW/TBW in the HBV Group and the HCV Group according to FIB-4 Index

Since age and ALT revealed to be significant factors in the multivariate analysis, we further compared ECW/TBW in the HBV-group and the HCV group according to FIB-4 index [22,23,24,25].

**Figure 5:**
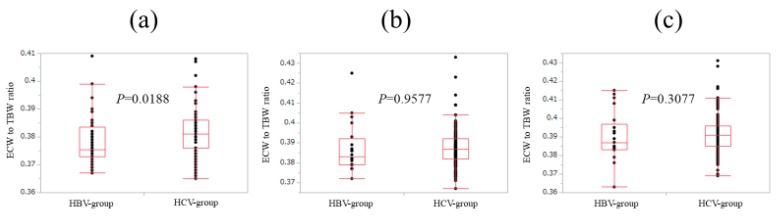
Comparison of ECW/TBW in the HBV group and the HCV group according to FIB-4 index. (**a**) In patients with FIB-4 index <2, ECW/TBW in the HCV group (*n* = 91) was significantly higher than that in the HBV group (*n* = 44) (median (range): 0.376 (0.367–0.409) in the HBV group and 0.381 (0.365–0.408) in the HCV-group, *p* = 0.0188). (**b**) In patients with 2 ≤ FIB-4 index <4, ECW/TBW in the HCV group (*n* = 139) was not significantly higher than that in the HBV group (*n* = 20) (median (range): 0.383 (0.372–0.425) in the HBV group and 0.387 (0.367–0.433) in the HCV group, *p* = 0.9577). (**c**) In patients with FIB-4 index ≥4, ECW/TBW in the HCV group (*n* = 210) was not significantly higher than that in the HBV group (*n* = 21) (median (range): 0.387 (0.363–0.415) in the HBV group and 0.391 (0.369–0.431) in the HCV group, *p* = 0.3077).

In patients with FIB-4 index <2 (*n* = 44 and 91 in the HBV and HCV groups), ECW/TBW in the HCV group was significantly higher than that in the HBV group (median (range): 0.376 (0.367–0.409) in the HBV group and 0.381 (0.365–0.408) in the HCV group, *p* = 0.0188) (Figure 5a).

In patients with 2≤ FIB-4 index <4 (*n* = 20 and 139 in the HBV and HCV groups), ECW/TBW in the HCV group was not significantly higher than that in the HBV group (median (range): 0.383 (0.372–0.425) in the HBV group and 0.387 (0.367–0.433) in the HCV group, *p* = 0.9577) (Figure 5b).

Likewise, in patients with FIB-4 index ≥4 (*n* = 21 and 210 in the HBV and HCV groups), ECW/TBW in the HCV group was not significantly higher than that in the HBV group (median (range): 0.387 (0.363–0.415) in the HBV group and 0.391 (0.369–0.431) in the HCV group, *p* = 0.3077) (Figure 5c).

## 4. Discussion

As far as we are aware, this is the first study comparing ECW/TBW between liver disease patients with HBV and HCV. The crucial barrier to improve fluid management is the limitation of identifying early or occult overhydrating, and thus identifying liver disease patients with overhydrated state in an earlier stage may lead to the adequate fluid management. We therefore conducted this comparative analysis.

In our results, the median (range) ECW/TBW in the two groups were 0.388 (0.365 to 0.433) in the HCV group and 0.381 (0.363 to 0.425) in the HBV group with statistical significance. In non-LC patients with FIB-4 index ≤2, ECW/TBW in the HCV group were significantly higher than those in the HBV group. These results denoted that the overhydrate state in the HCV group can occur in an earlier fibrotic stage (i.e., non-LC) than that in the HBV group and may provide useful information for clinicians in the fluid management of liver diseases patients. As reported earlier, in cases of healthy persons, EBW/TBW can be maintained at a constant value (0.38) and more than half of our non-LC subjects had EBW/TBW >0.38. These data also can be a point of focus.

One possible reason for the difference of ECW/TBW in HBV and HCV may be linked to baseline age difference in the two groups (median (range) age: 55.8 (28.8–77.0) in the HBV group and 65.0 (20.8–94.0) in the HCV group, *p* < 0.0001). Malczyk et al. [26] reported that ECW/TBW increased significantly with age, which is in line with our results (our data: *r_s_* (ECW/TBW and age) = 0.6085, *p* < 0.0001). Japanese liver disease patients are aging these days [27,28]. Elderly patients show various changes of the liver and other organs that could affect the clinical characteristics and management of liver diseases and caution should be exercised for the fluid management in elderly liver disease patients [29,30].

It is of note that for predicting F3 or more, ECW/TBW yielded the AUROC with a level of 0.74912 and for predicting F4, ECW/TBW yielded the AUROC with a level of 0.75517 in our data, which suggests its well predictability for liver fibrosis. Ianni et al. [31] reported that bioimpedance technology using delta of the electrical resistance values had good level sensitivity and acceptable specificity for detecting liver fibrosis. Thus, BIA can be helpful in various clinical aspects.

In our multivariate analysis, lower serum albumin, higher age, lower PT, and lower ALT were significantly associated with ECW/TBW ≥0.4. Albumin is the most abundant protein in extracellular fluid and accounts for about 70% of the plasma colloid osmotic pressure, and thus it plays an important role in regulating fluid distribution in the human body [32]. The significance of a lower ALT value may be attributed to LC with remission of chronic inflammation due to antiviral therapies. In our data, the proportions of ALT <33 IU/L in non-LC and LC patients were 43.95% (98/223) and 50.66% (153/302), respectively. On the other hand, advanced LC with overhydrated status can cause malnutrition and muscle wasting (sarcopenia) and lower SMI was expected to be associated with ECW/TBW ≥0.4, but actually it was not so. Overhydrated status may lead to the overestimation of skeletal muscle mass and this may cause the non-significance of SMI in the unilabiate analysis linked to ECW/TBW ≥0.4 [33,34]. 

We acknowledge several limitations to this study. First, our study was a retrospective single-center study; a larger prospective multi-center study is needed for further prospective external validation. Second, the number of HBV and HCV patients were not well balanced for analysis. Third, the study was based on a Japanese population, and additional studies on different ethnic backgrounds are necessary to further validate and extrapolate to non-Japanese backgrounds. Fourth, a number of patients received previous antiviral therapies, which potentially leads to bias. However, our data surely shed some light on the fluid management in viral liver diseases.

## 5. Conclusions

In conclusion, ECW/TBW in liver diseases can be different according to hepatitis viruses. Overhydrated status can easily occur in the HCV group as compared with the HBV group. Clinicians should be aware of these for the adequate fluid management in viral associated liver diseases and ECW/TBW in BIA can be helpful for predicting liver fibrosis.

## Figures and Tables

**Table 1:** Baseline data (*n* = 525).

Variables	All Cases (*n* = 525)	HCV Group (*n* = 440)	HBV Group (*n* = 85)	*p* Value (HCV vs. HBV)
Age (years)	64.4 (20.8–94.0)	65.0 (20.8–94.0)	55.8 (28.8–77.0)	<0.0001
Gender, male/female	268/257	218/222	50/35	0.1247
Body mass index (kg/m^2^)	22.2 (13.1–34.4)	22.2 (13.1–34.4)	22.2 (15.9–30.4)	0.6839
ECW to TBW ratio	0.387 (0.363–0.433)	0.388 (0.365–0.433)	0.381 (0.363–0.425)	<0.0001
Skeletal muscle mass index (cm^2^/m^2^)	6.56 (3.50–10.21)	6.51 (3.50–10.21)	6.92 (5.21–9.90)	0.0099
Liver histology (F0-1, F2, F3, Child-Pugh A/B/C)	106/53/64/226/72/4	79/41/56/198/62/4	27/12/8/28/10/0	0.0394
Total bilirubin (mg/dL)	0.9 (0.2–5.1)	0.9 (0.2–5.1)	0.9 (0.3–2.8)	0.5264
Serum albumin (g/dL)	3.9 (2.3–5.1)	3.9 (2.3–5.1)	4.0 (2.7–5.1)	0.0017
Prothrombin time (%)	87.0 (39.2–130.3)	86.4 (39.2–130.3)	89.6 (43.3–123.0)	0.2371
Platelet count (× 10^4^/mm^3^)	12.8 (2.6–48.1)	12.4 (3.0–48.1)	14.9 (2.6–37.9)	0.0022
Serum creatinine (mg/dL)	0.68 (0.30–7.69)	0.67 (0.30–7.69)	0.75 (0.46–6.0)	0.0021
Total cholesterol (mg/dL)	156 (73–293)	153 (73–293)	171 (82–293)	0.0100
Triglyceride (mg/dL)	85 (25–554)	85 (25–554)	85 (30–256)	0.5929
AST (IU/L)	38 (10–756)	40 (10–349)	30 (13–756)	<0.0001
ALT (IU/L)	33 (7–1079)	34 (7–258)	29 (7–1979)	0.0304
BTR	4.95 (1.65–19.68)	4.77 (1.65–19.68)	5.85 (1.82–11.28)	0.0002
FIB-4 index	3.56 (0.22–20.04)	3.80 (0.22–20.04)	1.95 (0.37–19.14)	<0.0001
Fasting blood glucose (mg/dL)	99 (64–403)	100 (64–403)	95 (70–283)	0.1764

Data are expressed as number or median (range). HBV: hepatitis B virus; HCV: hepatitis C virus; ECW: extracellular water; TBW: total body water; AST: aspartate aminotransferase; ALT: alanine aminotransferase; BTR: branched-chain amino acid to tyrosine ratio.

**Table 2:** Relationship between extracellular water to total body water ratio and baseline characteristics.

	*r_s_*	*p* Value
Age	0.6085	<0.0001
Body mass index	−0.0742	0.0896
Skeletal muscle index	−0.3059	<0.0001
Total bilirubin	0.0432	0.3228
Serum albumin	−0.4145	<0.0001
Prothrombin time	−0.4210	<0.0001
Platelet count	−0.3241	<0.0001
Serum creatinine	−0.1764	<0.0001
Total cholesterol	−0.2509	<0.0001
Triglyceride	−0.1980	<0.0001
AST	0.0478	0.2740
ALT	−0.2709	<0.0001
FIB-4 index	0.4915	<0.0001
BTR	−0.4076	<0.0001
Fasting blood glucose	0.0558	0.2025

AST: aspartate aminotransferase; ALT: alanine aminotransferase; BTR: branched-chain amino acid to tyrosine ratio.

**Table 3:** Comparison of baseline characteristics between the ECW to TBW ratio ≥0.4. Group and the ECW to TBW ratio <0.4 group.

Variables	ECW to TBW Ratio ≥0.4 (*n* = 52)	ECW to TBW Ratio <0.4 (*n* = 473)	*p* Value
Age (years)	73.0 (49.5–94.0)	63.2 (20.8–89.8)	<0.0001
Gender, male/female	22/30	246/227	0.1919
Cause of liver disease HBV/HCV	9/43	76/397	0.8428
Body mass index (kg/m^2^)	23.0 (16.5–31.8)	22.1 (13.1–34.4)	0.3275
Skeletal muscle index	6.46 (4.40–10.21)	6.58 (3.50–9.57)	0.5380
Total bilirubin (mg/dL)	0.9 (0.2–3.1)	0.9 (0.2–5.1)	0.3063
Serum albumin (g/dL)	3.2 (2.3–5.0)	3.9 (2.6–5.1)	<0.0001
Prothrombin time (%)	71.75 (39.2–118.8)	88 (43.3–130.3)	<0.0001
Platelet count (× 10^4^/mm^3^)	11.05 (3.0–37.9)	13.0 (2.6–48.1)	0.1081
Serum creatinine (mg/dl)	0.73 (0.35–4.51)	0.68 (0.3–7.69)	0.3141
Total cholesterol (mg/dl)	132 (73–266)	158 (82–293)	0.0002
Triglyceride (mg/dl)	72 (25–149)	88 (25–554)	0.0035
AST (IU/L)	39 (15–105)	38 (10–756)	0.7295
ALT (IU/L)	23.5 (10–82)	35 (7–1079)	<0.0001
BTR	3.75 (1.65–9.63)	5.06 (1.76–19.68)	0.0002
Fasting blood sugar (mg/dl)	98.5 (72–215)	99 (64–403)	0.3624
FIB-4 index	6.40 (1.16–19.14)	3.40 (0.22–20.04)	<0.0001

Data are expressed as number or median (range). HBV: hepatitis B virus; HCV: hepatitis C virus; ECW: extracellular water; TBW: total body water; AST: aspartate aminotransferase; ALT: alanine aminotransferase; BTR: branched-chain amino acid to tyrosine ratio.

**Table 4:** Significant factors in the multivariate analyses linked to extracellular water to total body water ratio ≥0.4.

Variables	Multivariate Analysis		
	Hazard Ratio	95% Confidence Interval	*p* Value
Age ≥64.4 years	3.921	1.802–8.526	0.0006
Serum albumin <3.9 g/dL	4.295	1.890–9.761	0.0005
PT <87.0%	2.607	1.089–6.241	0.0314
ALT <33 IU/l	4.143	2.059–8.336	<0.0001

PT; prothrombin time, ALT; alanine aminotransferase.

## Abbreviations

BIA	bioimpedance analysis
ECF	extracellular fluid
ECW	extracellular water
TBW	total body water
HBV	hepatitis B virus
HCV	hepatitis C virus
LC	liver cirrhosis
BMI	body mass index
SMI	skeletal muscle mass index
ROC	receiver operating characteristics
AUROC	area under the ROC curve
AST	aspartate aminotransferase
ALT	alanine aminotransferase
BTR	branched-chain amino acid to tyrosine ratio
PT	prothrombin time
